# Impacted Maxillary Canine Prevalence and Its Association with Other Dental Anomalies in a Mexican Population

**DOI:** 10.1155/2017/7326061

**Published:** 2017-02-23

**Authors:** José Rubén Herrera-Atoche, María del Rosario Agüayo-de-Pau, Mauricio Escoffié-Ramírez, Fernando Javier Aguilar-Ayala, Bertha Arelly Carrillo-Ávila, Marina Eduviges Rejón-Peraza

**Affiliations:** Facultad de Odontología, Universidad Autónoma de Yucatán, Mérida, YUC, Mexico

## Abstract

*Objective*. We quantified the prevalence of impacted maxillary canines (IMC) and their association with other dental anomalies (DAs).* Materials and Methods*. A retrospective study was done with 860 patients 12 to 39 years of age. The prevalence of IMC was calculated and compared by sex. The sample was divided into a control group and an impaction group, and the prevalence was calculated in both for a series of anomalies: agenesis, supernumerary teeth, shape anomalies of the upper laterals (microdontia, peg and barrel shape, and talon cusp), fusion, gemination, other impacted teeth, transposition, and amelogenesis imperfecta. The prevalence values for both groups were compared (Pearson's *χ*^2^ test, *p* ≤ 0.05).* Results*. IMC were present in 6.04% of the sample with no difference by sex (*p* = 0.540). Other DAs occurred in 51.92% of the IMC group and in 20.17% of the controls (*p* < 0.05). Significant associations (*p* < 0.05) were identified between IMC and four other DAs: microdontia, barrel shape, other impacted teeth, and transposition. The prevalence of all anomalies was lower in the control group.* Conclusion*. IMC were seen in 6.04% of patients. Patients with this condition also had a higher prevalence of other DAs. These other anomalies should be used as risk indicators for early diagnosis.

## 1. Introduction

A tooth is considered impacted when its eruption is hampered by other teeth, bone, or soft tissues [1]. Diagnosis is generally based on clinical and X-ray analyses [2]. Reported prevalence of impacted teeth in Mexico is 13.58% [3] making it the most common dental anomaly after agenesis (26-27%, third molars included) [4, 5].

If third molars are excluded, the maxillary canines are the most common teeth to experience impaction [3, 6–8], and patients with impacted maxillary canines (IMC) often exhibit other associated dental anomalies [9, 10]. The frequent association of these anomalies has led some researchers to suggest their use as markers to indicate the need for further clinical and/or X-ray analyses to create early diagnosis [11]. The upper lateral incisor is normally used as a marker because congenital absence, microdontia, peg shape, and even incorrect position of this tooth are associated with impaction of the maxillary canines [11–13].

When diagnosed at an early age, the infant canine is extracted as a recommended preventative measure [14]. Rapid maxillary expansion [15] and cervical traction using headgear [16] have also been reported to assist with spontaneous eruption of these teeth. A lack of treatment can lead to a number of risks including resorption of the roots of neighboring teeth, cyst formation, and development of malocclusions [13, 17, 18]. The objective of this study was to quantify the prevalence of impacted maxillary canines and their association with other dental anomalies in a Mexican population.

## 2. Materials and Methods

This retrospective study was done using case/control design. The sample consisted of 860 orthodontic patients 12 to 39 years of age of whom 32.67% were male (*n* = 281) and 67.33% were female (*n* = 579). Potential cases were excluded for patients with previous orthodontic treatment, cleft lip or palate, or any syndrome. A tooth was considered impacted when other teeth, bone, or soft tissues interfered with its eruption in normal functional occlusion [1]. IMC was diagnosed with panoramic X-rays, clinical photographs, and study models from patient files.

The prevalence was then calculated including studies of sex parity. This total sample was divided into two groups: patients with IMC and a control without it. The prevalence of eleven dental anomalies (DAs) was calculated in both groups: 2 count anomalies (agenesis and supernumerary teeth); 6 shape anomalies, four of the upper laterals (microdontia, peg- and barrel-shaped, and talon cusp) as well as tooth fusion and germination; 2 eruption anomalies (impaction of teeth other than the maxillary canines and transposition); and 1 structural anomaly (amelogenesis imperfecta). The identification of each DA used published definitions [3].

### 2.1. Statistical Analysis

Imposing sex parity on IMC and dental anomaly prevalence in both groups was done using Pearson's *χ*^2^ test (*p* ≤ 0.05). Fisher's exact test was used when the expected counts in some of the table cells were 5 or less. Calculations were also made for the confidence intervals (CI = 95%) and the odds ratio (OR).

### 2.2. Ethical Considerations

Data were obtained from the patient files. No patient was unnecessarily exposed to radiation, and all patients signed a consent form authorizing the institution to use clinical records for research purposes. This study was approved by the Research Ethics Committee of the “Centro de Investigaciones Regionales Dr. Hideyo Noguchi” (CIRB-2013-0016).

## 3. Results

At least one IMC was present in 6.04% (*n* = 52) of the sample. Although the male/female ratio in the sample was 1 : 2.06, the rates were similar among males (6.76%; *n* = 19) and females (5.69%; *n* = 33). No association was identified by sex (*p* = 0.540).

A total of 65 IMC were identified: 50.77% (*n* = 33) were on the right side of the maxilla and 49.23% (*n* = 32) were on the left. Of the 52 patients affected, 25% (*n* = 13) exhibited bilateral impaction.

Of the two groups, 51.92% (*n* = 27) of the IMC patients had associated DAs and 20.17% (*n* = 163) of the control group had associated DAs (*p* < 0.0001). Significant associations were identified between IMC and four DAs: microdontia of upper laterals, barrel-shaped lateral incisors, other impacted teeth, and teeth in transposition (*p* < 0.0001). The prevalence of each dental anomaly with significant associations in the control group was less than the IMC group (Table 1).

## 4. Discussion

The 6.04% IMC prevalence in the cohort is within the 1.73 to 9.02% interval reported in other populations [6–9, 11, 19, 20]; the 5.43% rate reported in Hungary is the closest to our study [20]. Bilateral impaction was present in 25% of the IMC patients—this is similar to the 19.2% observed in the Greek population [6]. The overall IMC prevalence in Mexico is also similar to the population of Greece [3, 6] meaning that the similarity in bilateral impaction between these populations can be expected.

Females have IMC more often [7, 11], but there is no evidence in our data or in other reports even when the proportion was greater among females [6, 8].

Associations were identified between IMC and four other DAs. This coincides with previous reports [9, 10, 12], although most of these studies only evaluated palatally displaced canines. However, in a study of Chinese patients exhibiting palatally and lingually displaced IMC, other DAs were associated. Indeed, 47.5% of these IMC patients had other DAs, which is similar to our findings [10].

Microdontia and barrel shape in the upper lateral incisors were associated with IMC. Previous reports showed associations between microdontia and peg-shaped teeth with IMC [10, 12]. This study coincided with the association between microdontia and IMC, but barrel-shaped rather than peg-shaped incisors were the second association. This could be an artifact of the higher prevalence of microdontia and barrel-shaped teeth in Mexico compared to peg-shaped teeth in other populations [3]. Upper lateral incisor anomalies (e.g., agenesis, microdontia, and peg shape) should be seen as markers for IMC [12]. Our data suggests that the barrel shape should be included in this group of markers for a Mexican population.

The prevalence of IMC was also associated with the presence of other impacted teeth, which agrees with data from a Chinese population [10]. Associations between anomalies can often be explained genetically [21, 22] especially when the impacted tooth is far from the IMC in question. However, environmental factors can also cause this anomaly, for example, an impacted central incisor on the same side as an IMC. In this case, canine impaction can be the consequence of the impacted incisor, which would explain the rarity of these events occurring together [23]. Further research is needed to determine whether the cause of association between these anomalies is primarily genetic or environmental.

On the other hand, the risk of tooth transposition was extremely high in patients with IMC. This can easily be explained because the maxillary canines are the teeth that most frequently experience transposition [3, 24].

Of note, dental agenesis was among the anomalies that showed no statistical association even when in the past it had been reported that patients with impacted maxillary canines usually have associated DA [9, 10, 25–28]. In addition, some authors have highlighted maxillary incisor agenesis as a common clinical finding [10, 25–27]. However, research into a Mexican population showed no association between agenesis and impacted teeth [3]. Moreover, a study on patients with maxillary incisor agenesis in a Brazilian population found no significant association between impacted canines and the absence of these teeth [29]. Latin American populations do not show this phenomenon [3, 29]. Thus, this explanation may rely on ethnic differences. Thus, a similar study should be done on other populations before reaching broad conclusions.

## 5. Conclusions

Maxillary canine impaction occurs with a frequency of 6.04% in this population. Patients with IMC also had a higher prevalence of other dental anomalies—this increase was caused by the greater presence of microdontia and barrel-shaped lateral teeth as well as other impacted and transpositioned teeth. The presence of microdontia and/or barrel-shaped upper lateral teeth could be used as an IMC risk marker in this Mexican population.

## Figures and Tables

**Table 1:** Prevalence and distribution of dental anomalies by group; total sample size = 860.

Dental anomalies	IMC^1^		Control		*p*	OR^2^	CI^3^
	*n* = 52	6.04%	*n* = 808	93.95%			
Total prevalence	27	51.92%	163	20.17%	<0.0001^*∗*^	4.27	2.41–7.56
Agenesis	4	7.69%	39	4.82%	0.322	1.64	0.56–4.78
Supernumerary teeth	3	5.76%	40	4.95%	0.740	1.17	0.35–3.93
Microdontia	11	21.15%	37	4.57%	<0.0001^*∗*^	5.59	2.66–11.75
Peg-shaped upper laterals	1	1.92%	11	1.36%	0.529	1.42	0.18–11.22
Barrel-shaped upper laterals	7	13.46%	15	1.85%	<0.0001^*∗*^	8.22	3.19–21.18
Upper laterals with talon cusp	0	0%	16	1.98%	—	0.98	0.97–0.99
Fusion	0	0%	2	0.24%	—	0.99	0.99–1
Gemination	0	0%	1	0.12%	—	0.99	0.99–1
Impacted teeth	10	19.23%	45	5.56%	<0.0001^*∗*^	4.03	1.9–8.56
Transposition	12	23.07%	9	1.11%	<0.0001^*∗*^	26.63	10.6–66.88
Amelogenesis imperfecta	0	0%	2	0.24%	—	0.99	0.99–1

^*∗*^Statistically significant (*p* ≤ 0.05). ^1^IMC: impacted maxillary canine. ^2^OR: odds ratio. ^3^CI: confidence interval.
